# Melatonin for Insomnia in Medical Inpatients: A Narrative Review

**DOI:** 10.3390/jcm12010256

**Published:** 2022-12-29

**Authors:** Christine Salahub, Peter E. Wu, Lisa D. Burry, Christine Soong, Kathleen A. Sheehan, Thomas E. MacMillan, Lauren Lapointe-Shaw

**Affiliations:** 1 Support, Systems, and Outcomes Department, University Health Network, Toronto, ON M5G 2C4, Canada; 2 Division of Clinical Pharmacology & Toxicology, University of Toronto, Toronto, ON M5S 1A8, Canada; 3 Department of Medicine, University of Toronto, Toronto, ON M5S 3H2, Canada; 4 Department of Pharmacy and Medicine, Sinai Health System, Toronto, ON M5G 1X5, Canada; 5 Interdepartmental Division of Critical Care, Leslie Dan Faculty of Pharmacy, Toronto, ON M5G 3M2, Canada; 6 Department of Family and Community Medicine, University of Toronto, Toronto, ON M5S 2E8, Canada; 7 Division of General Internal Medicine and Geriatrics, University Health Network and Sinai Health System, Toronto, ON M5G 2C4, Canada; 8 Institute of Health Policy, Management and Evaluation, University of Toronto, Toronto, ON M5T 3M6, Canada; 9 Department of Psychiatry, University of Toronto, Toronto, ON M5T 1R8, Canada; 10 Centre for Mental Health, University Health Network, Toronto, ON M5T 2S8, Canada; 11 ICES, Toronto, ON M4N 3M5, Canada; 12 Women’s College Institute for Health System Solutions and Virtual Care, Women’s College Hospital, Toronto, ON M5S 1B2, Canada

**Keywords:** melatonin, insomnia, medical inpatient, sleep, general internal medicine, sedative hypnotic

## Abstract

In this narrative review, we describe what is known about non-pharmacological and pharmacological treatments for insomnia in medical inpatients, with a focus on melatonin. Hospital-acquired insomnia is common, resulting in shortened total sleep time and more nighttime awakenings. Sleep disturbance has been shown to increase systemic inflammation, pain, and the likelihood of developing delirium in hospital. Treatment for insomnia includes both non-pharmacological and pharmacological interventions, the latter of which requires careful consideration of risks and benefits given the known adverse effects. Though benzodiazepines and non-benzodiazepine benzodiazepine receptor agonists are commonly prescribed (i.e., sedative-hypnotics), they are relatively contraindicated for patients over the age of 65 due to the risk of increased falls, cognitive decline, and potential for withdrawal symptoms after long-term use. Exogenous melatonin has a comparatively low likelihood of adverse effects and drug–drug interactions and is at least as effective as other sedative-hypnotics. Though more research is needed on both its effectiveness and relative safety for inpatients, small doses of melatonin before bedtime may be an appropriate choice for inpatients when insomnia persists despite non-pharmacological interventions.

## 1. Case Example

An 84-year-old woman is admitted to hospital after suffering a fall. She reports poor sleep on her first night due to evening vital checks, noise at the nursing station, and an uncomfortable bed. She is given more pillows, an eye mask, and earplugs. On night two, the patient again reports being unable to sleep despite non-pharmacological interventions and requests a “sleeping pill”. The attending physician prescribes her 5 mg of zolpidem and, the next morning, the patient reports having slept for 6 h. The patient stays in the hospital for a week and is discharged home with a new prescription for zolpidem with instructions to use only when needed. A week later, shortly after waking, she has another fall resulting in a hip fracture. Her family wonders whether there was some contribution of the sleeping pill, as she had been taking it daily to help her fall asleep and reported feeling drowsy in the mornings.

## 2. Introduction

Hospital inpatients often report poor sleep quality and quantity, which is influenced by many factors, including noisy environments, clinical-care interruptions, uncomfortable beds, bright lighting, unfamiliar surroundings, medication adverse effects, illness, anxiety, and pain [1,2,3]. Although hospitalization-related insomnia is often an acute phenomenon [4], those suffering from chronic insomnia may also experience an exacerbation of symptoms while hospitalized. Compared with sleep at home, inpatients have shorter sleep duration by more than an hour, wake up on average 44 min earlier, and have more nighttime awakenings [5]. Inadequate sleep can have negative physiologic and psychological effects, including systemic inflammation [6,7], increased pain [8,9], anxiety [10], insulin resistance [11], and delirium [12,13]. Given the importance of sleep to patients’ overall health and well-being, finding effective interventions is a clinical and research priority.

Although non-pharmacological interventions are the recommended first line of treatment for insomnia [7,14,15], the evidence for their effectiveness in hospital is mixed [7,16], with the most effective interventions consisting of a comprehensive/multi-modal approach [17]. Benzodiazepines and non-benzodiazepine γ-aminobutyric acid receptor agonist agents (z-drugs) are commonly prescribed to inpatients [1,18,19,20]. These two types of drugs, which we will refer to as sedative-hypnotics, are not recommended for those over the age of 65, due to an increased risk of falls and development of delirium [21,22]. The commonly used over-the-counter supplement, melatonin, is not contraindicated in older adults and has equal or lower incidence of adverse events compared to sedative-hypnotics [23,24].

The objective of this paper is to present a narrative review of non-pharmacological and pharmacological treatments for insomnia in medical inpatients, with a focus on melatonin. We propose that prior to initiating a new sedative-hypnotic, physicians should consider whether their patient would benefit from a trial of melatonin in addition to good sleep-hygiene practices (see Table 1 for a summary of the literature and recommendations for melatonin use).

## 3. Non-Pharmacological Interventions for Insomnia in Hospital Inpatients

As in all medicine, clinicians should preferentially choose the treatment with the least associated harm. Given their relative safety compared to pharmacotherapy, non-pharmacological interventions are recommended as first-line treatment for inpatient insomnia [13,14,38]. Interventions include the use of relaxation techniques [14], increasing daytime bright-light exposure [39,40,41], providing earplugs/eye masks [16], and reducing nighttime nursing care activities [16,42]. A multi-modal approach should be considered, including taking a thorough sleep history, reviewing prescriptions, treating comorbid conditions/symptoms that may be interfering with sleep (e.g., pain/anxiety/nausea), and psychoeducation about sleep habits and attitudes [43]. In a 2014 meta-analysis of sleep interventions for inpatients, it was found that relaxation techniques, better sleep hygiene, and daytime bright-light exposure were each effective for improving sleep quality and/or quantity [14]. A more recent meta-analysis (2019) came to similar conclusions, but also found cognitive behavioral therapy for insomnia (CBT-I) to be an effective intervention [7,44]. CBT-I interventions combined with stimulus control, sleep restriction, sleep hygiene, and/or relaxation techniques were found to improve sleep onset latency by 19 min and sleep efficiency by 9.9%, with no adverse outcomes [45]. Though many patients prefer non-pharmacological treatments over sedative-hypnotic medications [46], they are sometimes not aware of these options due to implementation barriers, such as healthcare provider time constraints [38,47], resulting in underutilization of non-pharmacological approaches.

It is difficult to ascertain from the available literature which non-pharmacological interventions are most effective due to research limitations, such as failure to use objective sleep measures, medium to high risk of bias (i.e., sampling error or performance bias), and lack of control groups [7].

## 4. Pharmacological Sleep Aids for Inpatient Sleep

Recent developments in pharmacological therapies for insomnia have given prescribers more options. Dual orexin receptor antagonists are safe, effective, and approved to treat primary insomnia; these may not yet be present on hospital formularies, as they are relatively new and costly [48,49]. Low-dose doxepin, a tricyclic antidepressant and selective histamine antagonist, has also been found to be safe for the treatment of insomnia (an off-label use) in older adults [50,51], although it also may not be widely available on hospital formularies. Neither dual orexin receptor antagonists nor low-dose doxepin have been studied in hospital inpatients. 

Benzodiazepines (e.g., lorazepam) and non-benzodiazepine γ-aminobutyric acid receptor agonist agents (z-drugs, e.g., zopiclone, zaleplon, eszopiclone, and zolpidem tartrate) are the most commonly prescribed sedative-hypnotics for hospital-acquired insomnia [1,18,20]. Despite the widespread use of sedative-hypnotics, there is inconsistent evidence that they improve sleep quality or quantity in inpatients. In a hospital setting, benzodiazepines have been found to shorten sleep latency but have little effect on total sleep time or sleep quality [20,52]. Interestingly, one systematic review found that sedative-hypnotics are equally effective not only to each other, but also to placebo or no treatment at all [28].

Given the increased risk of falls and hip fractures with sedative-hypnotic use [21,29,53,54], there is a broad consensus that these medications should be avoided in adults over the age of 65; they are included on the American Geriatrics Society Beers Criteria^®^ list of potentially inappropriate medications, and the Choosing Wisely guidelines also recommend against their use [22,55]. In older patients, long-term use of benzodiazepines has also been found to increase the risk of developing cognitive decline, dementia, or delirium [56,57,58,59]. Z-drugs have similar risks and are, therefore, recommended for short-term use only and not in combination with benzodiazepines [22]. Gillis et al. (2014) [18] found that most hospitalized patients who received a medication for sleep had no prior history of insomnia or use of pharmacological sleep aids (68.5%) and that 34.4% were discharged with a new prescription. There is evidence that new benzodiazepine prescriptions lead to continuing use in some patients [60]. After prolonged use, attempts to wean these medications can cause withdrawal symptoms, such as insomnia, anxiety, and tremors [61,62]. 

Other medications, such as antipsychotics (e.g., quetiapine, risperidone), antihistamines (e.g., diphenhydramine, dimenhydrinate), and anti-depressants (e.g., mirtazapine, trazodone, amitriptyline), are occasionally used as off-label sleep aids in hospitalized patients [19,20]. In older adults with dementia, atypical antipsychotic use has been associated with an increased risk of stroke [63] and death [64]. Antihistamines with anticholinergic activity can cause confusion and are, therefore, listed as drugs to avoid on the Beers Criteria^®^ list for medication use in older adults [22,65]. Sedating anti-depressants may not be any safer [66], as they also come with significant side effects, including risk of hypotension [67] and falls [68]. Some have anticholinergic side effects, such as urinary retention and confusion, and many have the potential for serious drug–drug interactions [69]. 

Together, these findings call into question whether the use of prescription sedative-hypnotics in hospital inpatients is warranted, particularly for older individuals [70]. Furthermore, sedative-hypnotic prescriptions may contribute to drug diversion and recreational use [71], and interactions with other drugs (e.g., opioids) can potentially be fatal [72].

## 5. Melatonin Supplementation for Outpatient Sleep

Melatonin is the primary regulatory hormone of the circadian rhythm in humans [25,73,74]. It is secreted by the pineal gland and synthesized from serotonin, which originates from tryptophan, a natural mild sedative [25,75,76,77]. Melatonin peaks in low light and is suppressed by daylight; the onset of its secretion positively correlates with sleepiness [73,78]. There are large interindividual differences in the timing of peaks and serum concentration levels of endogenous melatonin [74], with an average plasma concentration between 20 and 70 pg/mL [30,74]. In healthy adults, the natural peak concentration of melatonin has been found to vary more than 10-fold between individuals, with an average peak of around 60 pg/mL between 2 and 4 a.m. [75,79]. Peak nocturnal melatonin concentrations decline with age (65–70 years: 49.3 pg/mL, ≥75 years: 27.8 pg/mL) [80], and delayed peaks are associated with sleep disorders in older adults [81].

Due to its chronobiotic properties, exogenous melatonin is used to treat a variety of sleep conditions associated with shifted or abnormal circadian clocks [26,82], such as insomnia [83], jetlag [84], and shift-work syndrome [26], despite inconsistent evidence for sleep improvement [26,85]. In people with or without insomnia, melatonin reduces sleep onset latency and increases total sleep duration, though it is unknown whether these changes are clinically meaningful [73,86]. In one small study (41 older outpatients without dementia), melatonin improved sleep quality and next-day function, while reducing benzodiazepine use and the number of nighttime awakenings [87].

Over-the-counter melatonin is considered a dietary supplement and comes in a variety of forms, including liquid, rapid-dissolve tablets and strips, gel and solid tablets, and compounds with other vitamins and minerals [35]. Doses of oral melatonin are typically between 2 mg and 10 mg daily, but doses as low as 0.3 mg were found to be effective when administered in the early evening [34,36,88]. Since doses of 10 mg were found to increase melatonin concentrations to as high as 14,000 pg/mL, lower doses may be more appropriate and prevent carryover daytime sleepiness [89].

In part due to its over-the-counter status, melatonin is one of the most widely used sleep aids [73,75,90]. Additionally, in the US, ramelteon is a synthetic melatonin receptor agonist (8 mg tablet) approved by the Food and Drug Administration for the treatment of insomnia in 2005 [27,91]. In animal cell studies, ramelteon’s affinity for melatonin receptors type 1 and type 2 was 6- to 13-times higher than melatonin [92]. This drug can be used for any length of time and is not a controlled substance; there is no evidence to suggest a potential for abuse/dependence [27].

## 6. Melatonin for Inpatient Sleep

In contrast with the literature for outpatients, few studies have examined the use of exogenous melatonin for inpatient sleep. A recent assessment by Macmillan et al. [19], in two Toronto hospitals, found that between 2013 and 2018, melatonin use increased from nearly no doses to more than 70 doses per 1000 patient days, whereas zopiclone use decreased over this time. White et al. [20] found that melatonin was the most-prescribed medication for hospital inpatient sleep (ICU and medical, 70.5%), followed by benzodiazepines (9.6%). This is likely due to increased recognition of the potential harms of sedative-hypnotics and low incidence of adverse effects and drug–drug interactions with melatonin [20]. Despite the increase in melatonin prescriptions for hospitalized patients, few studies have examined the impact of melatonin on sleep outcomes in general medical inpatient wards [93,94]. In a meta-analysis of two randomized-controlled trials (RCTs) in inpatients, melatonin and ramelteon use was associated with improved sleep quality, longer average hours of night sleep, and fewer nighttime awakenings [31].

More studies have examined melatonin use in ICU and post-operative settings. In two RCTs in the ICU, exogenous melatonin (10 mg) increased sleep by one hour and improved sleep quality (as evaluated by subjective questionnaires and electroencephalographic recordings) compared to placebo [30,89]. Although the limited evidence does suggest that exogenous melatonin alters sleep parameters, it is unknown whether these small differences are clinically meaningful, in particular whether or not they benefit patients in other ways (e.g., time to recovery or hospital discharge, incidence of delirium).

## 7. Melatonin and Delirium

Delirium is a common condition that affects at least 10% of inpatients over the age of 65 [95] and up to 70% in an ICU setting [12,96,97]. This syndrome results in acute changes in levels of consciousness and abnormal behavior, which typically lasts between 48 and 72 h, but often persists much longer [98]. Although the pathogenesis of delirium is not fully known, dysregulation of the sleep–wake cycle is a key feature of the condition [99,100]. Delirium prolongs hospital length of stay, has high associated mortality, and can lead to more permanent cognitive decline in survivors of critical illness [97,101,102].

Due to its role in regulating the circadian rhythm, melatonin has been used to prevent delirium in older hospitalized patients [101]. In patients with delirium, serum levels of melatonin are reduced [103] and circadian melatonin secretion is desynchronized or absent [104]. For elderly patients on hospital medical wards, a systematic review of two studies [93,95] reported that 0.5 mg to 5 mg of exogenous melatonin/ramelteon decreased the incidence of delirium by 75% compared to placebo [103]. A more recent systematic review excluded one of the prior studies and added a more recent trial and concluded that there was no effect of melatonin on delirium for medical inpatients, yet it reduced delirium in surgical and ICU patients [31].

A recent large, multicentre ICU trial found no significant difference in delirium incidence between melatonin (4 mg) and placebo groups [32]. The authors of an accompanying Editorial state that the most effective interventions have been multi-faceted; hence, there is a need to determine whether melatonin has any benefit over and above multi-component delirium prevention interventions [33,105]. Although it is unclear whether or not melatonin prevents delirium, the evidence indicates that it does not promote delirium over and above placebo, in contrast to benzodiazepines [56]. There is evidence that ramelteon may have a stronger effect on delirium prevention than melatonin, which could be explained by its stronger melatonin type 1 and type 2 receptor binding affinity [31,92].

## 8. Melatonin Compared to Sedative-Hypnotics

In most studies, melatonin and ramelteon have been compared to placebo. Unfortunately, few have directly compared sedative-hypnotics to melatonin. In a small (*N* = 14) cross-over study of healthy individuals, exogenous melatonin (0.1 mg to 10 mg) increased total sleep time, sleep efficiency, and amount of deep sleep compared to placebo [34]. This effect was similar to that obtained with 20 mg of temazepam and was present at 0.5 mg of melatonin.

In one study of 100 hospitalized patients (96% medical/surgical, 4% ICU), melatonin (3 mg to 10 mg) was compared with zolpidem 5 mg or 10 mg [24]. No significant differences were found in the number of self-reported sleep disturbances, sleep effectiveness, or sleep supplementation (i.e., needing a nap during the day) between the two medications [24]. Both groups experienced some headache and grogginess, but overall adverse event rates were low (10%). These findings suggest that from the patient’s perspective, melatonin and zolpidem have similar effects on sleep. As this was a single-centre study of 100 patients, more research is needed to determine the safety and efficacy of melatonin compared to other sedative-hypnotics in the inpatient setting.

## 9. Concerns about Melatonin

Healthcare providers may remain skeptical about the benefits of melatonin [106,107]. This could be due to its unregulated use, as it is licensed as a health product and not as a drug. Melatonin content has been found to be highly variable between brands and even between lots within the same brand [35]. Additionally, unlabeled serotonin has been found in some melatonin supplements, raising a theoretical concern for serotonin syndrome [35]. There are also concerns about the stability of liquid melatonin with different pH levels, elevated temperatures, and light exposure [108]. There is an opportunity for hospitals to play a leading role in sourcing reliable melatonin supplements that adhere to high standards of quality control, ensuring that labeled doses reflect true contents, without contamination by other active agents. 

Guidelines for melatonin dosage and timing of administration are lacking, especially for hospitalized patients. Doses provided in the early evening are more effective at shifting sleep earlier and, therefore, increasing total sleep time [34,74]. Given that there are high interindividual differences in melatonin bioavailability, dosing can be complex [109]. These uncertainties suggest that doses should start low (1 mg) and be increased by small increments. There is little evidence of any additional benefit from doses above 3 mg [34,49,73,88,110]. 

More research is needed into the interaction of melatonin with other drugs. Most reported side effects of melatonin in the general population are minor and short-lived, such as daytime sleepiness, headache, dizziness, or mood changes [36,90]. In a systematic review of clinical studies, adverse drug events from melatonin were unlikely to occur (reported in 24 out of 50 studies) and most were associated with daytime dosing, very high doses, or in specific patient populations [36]. One such population was patients receiving nifedipine for hypertension, for whom melatonin (5 mg) had mixed effects on blood pressure and heart rate [36,111]. Notably, the nighttime dosing and a short duration of action (i.e., maximal plasma/serum concentration ~50 min after oral dose, half-life of ~45 min [112]) of exogenous melatonin is theoretically advantageous, as effects should dissipate before morning. Therefore, based on current evidence, melatonin has an overall favorable safety profile [113].

Melatonin side effects are frequently milder than those of sedative-hypnotics and are often not significantly different from placebo [36]. For example, in healthy individuals aged 55 and older, zolpidem was found to impair psychomotor, driving performance, and memory recall up to 12 h post-administration, whereas prolonged-release melatonin did not impair performance in any of these tasks [23]. Melatonin, however, should not be mixed with sedatives, as this can amplify cognitive impairment [23].

There are few studies on the effects of long-term melatonin use, especially in older adults [113]. One study examined the 6- and 12-month efficacy and safety of prolonged-release melatonin (2 mg) in 112 adults with insomnia (age 20 to 80), followed by a 2-week withdrawal period [37]. There was no evidence of drug tolerance, adverse events, or withdrawal symptoms after 12 months compared to placebo. Additionally, there was no difference in the rate or types of adverse events between individuals younger and older than age 55. Similar effects have been observed with ramelteon (8 mg) after 6 months [114]. However, medical inpatients are typically older and have a higher level of medical complexity than the populations studied. Further research is needed to determine whether long-term use of melatonin by medical inpatients is associated with greater adverse events in hospital and/or following discharge.

## 10. Conclusions

Achieving high-quality, sufficient sleep is valued by patients, contributes to improved patient outcomes, and should be a clinical and research priority. Yet, it is often impaired in the inpatient setting. Sedative-hypnotics are commonly prescribed, but increase the risk of delirium, falls, and cognitive impairment, particularly in older individuals. In an inpatient setting, multi-modal non-pharmacological interventions should always be the first line of treatment. If these are insufficient, we suggest the addition of melatonin rather than sedative-hypnotics. Melatonin has a comparatively favorable safety profile and its equivalent effect on sleep quality and quantity make it a better choice.

## Figures and Tables

**Table 1:** Factors to consider when choosing a sleep aid for medical inpatients.

Factor	Summary of Literature	Key Papers	Recommendation
Melatonin for sleep in the general population	Low doses of melatonin are widely used in the general population for insomnia, jetlag, and shifted sleep. However, there is inconsistent evidence that it improves sleep.	Arora & Stewart (2018) [13]	Try non-pharmacological interventions first. If insomnia persists, exogenous melatonin can be added, but it may not be effective on its own.
		Hardeland et al. (2006) [25]	
		Costello et al. (2014) [26]	
		Neubauer (2008) [27]	
Pharmacological sleep aids for inpatient sleep	Benzodiazepines and z-drugs are the most commonly used sedative-hypnotics for hospital-acquired insomnia. Many patients are discharged with a new prescription. In patients over the age of 65, these drugs have been associated with increased fall risk and cognitive decline. Low-dose doxepin and dual orexin receptor antagonists have been found to be safe and effective in older adults, however, they have not been studied in hospitalised patients.	White et al. (2021) [20]	Avoid prescribing benzodiazepines and z-drugs, in particular for patients older than 65.
		Kanji et al. (2016) [28]	
		Finkle et al. (2011) [29]	
Melatonin for inpatient sleep	Melatonin use in hospital has increased in recent years, but few studies have examined use in medical inpatients. There is some evidence that it improves sleep quality and quantity, however more research is needed due to heterogeneity of the literature.	Macmillan et al. (2020) [19]	Exogenous melatonin may not be effective on its own for inpatient sleep, however, low doses are unlikely to be harmful.
		Gandolfi et al. (2020) [30]	
		Khaing & Nair (2021) [31]	
Melatonin and the prevention of delirium	There is inconsistent evidence for the effectiveness of exogenous melatonin in preventing delirium in medical inpatients.	Wibrow et al. (2022) [32]	For patients at risk of developing delirium, melatonin may be a safer choice than sedative-hypnotics. However, non-pharmacological delirium prevention interventions are preferred.
		Burry et al. (2021) [33]	
Melatonin vs. sedative-hypnotics	Melatonin has a similar effect as temazepam and zolpidem on sleep and is most effective when taken a couple of hours before bed. Overall adverse event rates in inpatients are low, with the most common side effects being headache and grogginess the following day.	Stoianovici et al. (2021) [24]	Small doses of melatonin are recommended and equally effective as temazepam. Administer in the early evening to be most effective.
		Stone et al. (2000) [34]	
Concerns about melatonin	Formularies: The content of melatonin supplements can be variable and include unlabeled serotonin. Guidelines for dosage are lacking and there are high interindividual differences in bioavailability.	Erland & Saxena (2017) [35]	Formularies: There is a need for consistent hospital formularies of exogenous melatonin from reliable sources.
	Drug Interactions: There may be interactions with antihypertensive medications (nifedipine).	Foley & Steel (2019) [36]	Drug Interactions: Monitor blood pressure and heart rate in patients on nifedipine due to the potential for melatonin to impair the antihypertensive efficacy of nifedipine.
	Long-Term Use: There is no evidence of long-term tolerance, adverse events, or withdrawal after 12-months.	Lemoine et al. (2011) [37]	Long-Term Use: Low doses of melatonin can likely be used long-term with no adverse effects, even in community-residing older adults.
